# The Microphenotron: a robotic miniaturized plant phenotyping platform with diverse applications in chemical biology

**DOI:** 10.1186/s13007-017-0158-6

**Published:** 2017-03-01

**Authors:** Thomas Burrell, Susan Fozard, Geoff H. Holroyd, Andrew P. French, Michael P. Pound, Christopher J. Bigley, C. James Taylor, Brian G. Forde

**Affiliations:** 1 Engineering Department, Lancaster University, Lancaster, LA1 4YR UK; 2 Lancaster Environment Centre, Lancaster University, Lancaster, LA1 4YQ UK; 3 The Centre for Plant Integrative Biology, School of Biosciences, Sutton Bonington Campus, University of Nottingham, Nottingham, LE12 5RD UK; 4 School of Computer Science, University of Nottingham, Jubilee Campus, Nottingham, NG8 1BB UK

**Keywords:** *Arabidopsis thaliana*, Automated, Biostimulants, Chemical biology, Chemical genetics, *Eragrostis tef*, Plant phenotyping, Robotic, Root system architecture, Shoot development

## Abstract

**Background:**

Chemical genetics provides a powerful alternative to conventional genetics for understanding gene function. However, its application to plants has been limited by the lack of a technology that allows detailed phenotyping of whole-seedling development in the context of a high-throughput chemical screen. We have therefore sought to develop an automated micro-phenotyping platform that would allow both root and shoot development to be monitored under conditions where the phenotypic effects of large numbers of small molecules can be assessed.

**Results:**

The ‘Microphenotron’ platform uses 96-well microtitre plates to deliver chemical treatments to seedlings of *Arabidopsis thaliana* L. and is based around four components: (a) the ‘Phytostrip’, a novel seedling growth device that enables chemical treatments to be combined with the automated capture of images of developing roots and shoots; (b) an illuminated robotic platform that uses a commercially available robotic manipulator to capture images of developing shoots and roots; (c) software to control the sequence of robotic movements and integrate these with the image capture process; (d) purpose-made image analysis software for automated extraction of quantitative phenotypic data. Imaging of each plate (representing 80 separate assays) takes 4 min and can easily be performed daily for time-course studies. As currently configured, the Microphenotron has a capacity of 54 microtitre plates in a growth room footprint of 2.1 m^2^, giving a potential throughput of up to 4320 chemical treatments in a typical 10 days experiment. The Microphenotron has been validated by using it to screen a collection of 800 natural compounds for qualitative effects on root development and to perform a quantitative analysis of the effects of a range of concentrations of nitrate and ammonium on seedling development.

**Conclusions:**

The Microphenotron is an automated screening platform that for the first time is able to combine large numbers of individual chemical treatments with a detailed analysis of whole-seedling development, and particularly root system development. The Microphenotron should provide a powerful new tool for chemical genetics and for wider chemical biology applications, including the development of natural and synthetic chemical products for improved agricultural sustainability.

**Electronic supplementary material:**

The online version of this article (doi:10.1186/s13007-017-0158-6) contains supplementary material, which is available to authorized users.

## Background

Chemical biology is the area of study where small molecules are used to perturb, investigate and control biological processes, while chemical genetics is a sub-field of chemical biology where the goal is primarily to investigate gene function [1, 2]. Although chemical genetics has become an important tool in many areas of research, and in the development of pharmaceuticals and agrochemicals [3–5], it has been less enthusiastically adopted by plant researchers, with some notable exceptions (e.g. [6–10]). This is despite the number of advantages that chemical genetics offers over conventional genetic approaches [1, 2], most notably its ability to overcome the problem of functional redundancy that can prevent single gene mutations having a discernible effect on the phenotype [11].

It is likely that one key reason for the low engagement with chemical genetics in plants is the limited range of plant phenotypes that it has been possible to screen for in a high-throughput format. The testing of tens of thousands of small molecules for their effect on the plant phenotype in a high-throughput manner is constrained by a number of factors. Firstly, to minimise the quantity of each small molecule that is needed and the physical space taken up by the experiment, assays are ideally performed in 96- (or 384-well) microtitre plates. Secondly, to avoid microbial degradation or other chemical modification of the small molecules before they can be absorbed by the plant, the assays are best performed under aseptic conditions. Because of its small size and its status as the pre-eminent model plant, *Arabidopsis thaliana* has been the species of choice for chemical genetic screens, and most screens have involved growing seedlings for a few days in 96-well microtitre plates, either in agar or liquid suspension (e.g. [9, 12–14]). However, under these conditions the space available for seedling development is very restricted and the ability to visualise and quantify growth and development within the wells, particularly of the root system, is necessarily very limited. Alternative approaches have used plant cell cultures [15] or germinating pollen [16], but again severely restricting the range of traits that it is possible to screen.

Recently a method for growing Arabidopsis seedlings in a 96-well format suitable for chemical genetic screens was described that for the first time allowed the detailed visualization of root development [17]. This technique was developed to make it possible to screen for small molecules that acted as antagonists of glutamate’s effect on root system architecture [18]. Briefly, the method involved the use of commercially available strips of 8 microtubes (FrameStrips™) filled with solid nutrient medium. When seed was sown on the gel surface, the growth and development of the root system could be visually monitored through the transparent walls of the tubes. Chemical treatments were applied through the excised ends of the microtubes by placing the strips into the wells of a microtitre plate and allowing the chemicals to diffuse upwards through the gel. This system was successfully used to identify two distinct classes of glutamate antagonists in a screen of 1576 yeast bioactives, leading to the identification of the MEKK1 MAP kinase kinase kinase as a component of the glutamate signalling pathway in Arabidopsis roots [17]. While this provided proof-of-principle for this miniaturised screening technique, its capacity for higher throughput applications is strictly limited, because the curved shape of the tubes and the 3-D pattern of root development within the tubes are not compatible with automated image capture and analysis.

In this paper we describe the development of an automated, high-content phenotyping platform for Arabidopsis seedlings that is designed to allow detailed observations of the development of root system architecture and its response to chemical treatments. In its current form this platform (the ‘Microphenotron’) has a capacity of up to 4320 individual chemical treatments in a typical 10 days experiment and the ability to robotically capture images on a daily basis for dynamic analysis of whole-seedling development. Phenotypic analysis can be performed either by visual inspection of images or by the use of specially developed software [19] that is able to automatically extract quantitative data on root and shoot development and shoot colour. We describe the use of the Microphenotron to screen a collection of 800 natural compounds for qualitative effects on root development, based on visual detection of alterations in nine root traits, leading to the identification of 70 bioactive molecules, most of which had not previously been reported to modify root development. In addition we describe the use of the Microphenotron to study the effects of a range of concentrations of nitrate and ammonium on seedling development using images captured daily and automated image analysis to determine the dynamics of the effects on root system development and shoot growth.

## Methods

### Plant material and growth conditions

The wild-type ecotype of *A. thaliana* L. was Col-0 and transgenic reporter lines were *DR5::GUS* [20] and *ARR5::GUS* (NASC N25261) [21]. Seed of *Eragrostis tef* (teff, var. Tsedey, DZ-Cr-37) was a gift from Dr. Terihun Tadele, University of Bern. All seed was surface-sterilized by rinsing briefly with absolute ethanol, followed by 10 min in sodium hypochlorite (2.8% available chlorine) and five washes with sterile deionised water. Before sowing, the seed was kept immersed in sterile deionised water in the dark at 4° for at least 2 days (Arabidopsis seed could be kept for up to a week under these conditions before sowing). Growth room temperatures of 21 ± 1° were maintained using a ceiling-located air conditioning unit for cooling, supplemented during the 8 h dark period with a free-standing domestic heater to replace the heat from the lights. No humidity control was required in the room because the seedlings were enclosed in growth boxes to maintain aseptic conditions and humidity inside the box was maintained with a blotting pad and a reservoir of water underneath the microtitre plate (see below under ‘Plate-holders’). Details of the lighting units are provided below in the section on "Supporting structure and lighting". The standard solid nutrient medium was based on 1/20th strength Gamborg’s B5 medium (pH 5.7) [22] containing 0.5 mM KNO_3_, 0.5% sucrose and 0.7% Phytagel with the addition of 1 mM MgCl_2_ and 1 mM CaCl_2_ to enable the Phytagel to set. Liquid medium in the microtitre plates had the same composition except for the absence of Phytagel and the additional MgCl_2_ and CaCl_2_.

### Preparation of the assay plates and seed sowing

**Fig. 1:**
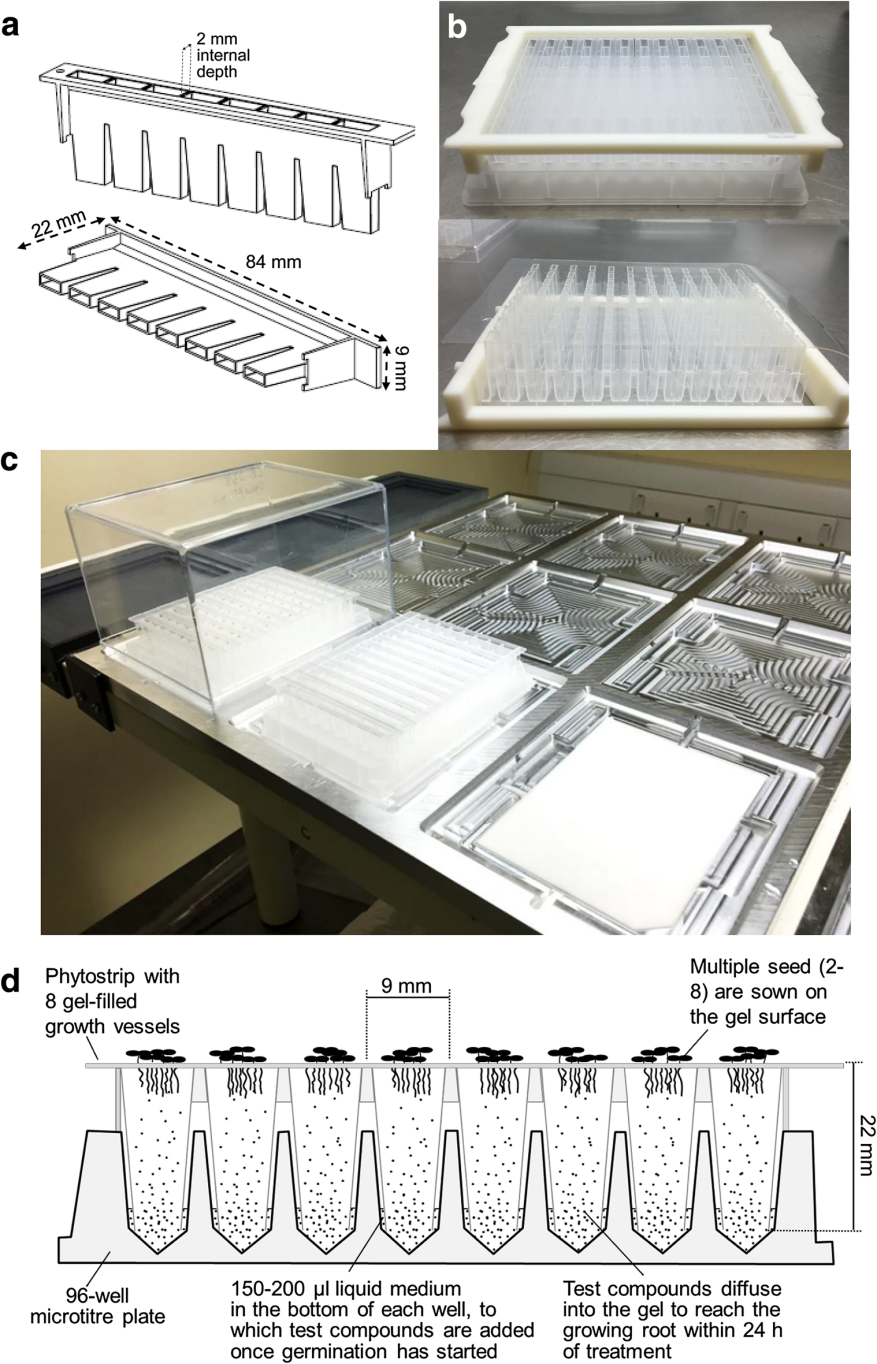
The Phytostrip growth device and stages in setting up the assay plates. **a** Line diagram showing two views of the custom-made Phytostrip. The Phytostrips (registered EU Community design no. 002115956) are manufactured in clear polypropylene to allow roots to be readily imaged. The wells of the Phytostrip are 22 mm in length and taper from *top* to *bottom*; the internal dimension of each well from front to back is uniformly 2 mm. **b** A set of 12 Phytostrips resting in a 96-well microtitre plate and held together with a custom-made clamping device to allow them to be moved as a block (*top*) and then inverted and sealed at the base with adhesive film (*bottom*) so that they can be filled with molten Phytagel medium. **c** Image showing three stages (from *right* to *left*) in the assembly of the assay plates in their plastic growth boxes. Each of the nine positions in a plate-holder has three separate sections with different layers of indentation (see Additional file 1: Figure S2). The innermost and lowest layer is a reservoir which holds a moistened blotting pad and is kept topped up with water to maintain humidity around the assay plates. The middle layer holds the assay plate in position above the blotting pad. The upper layer provides a holder for the clear plastic growth box that completes the assembly. The plate-holder is located in a fixed position on the robotic plinth by angle brackets so that the robotic arm can accurately locate each Phytostrip by its x, y coordinates. **d** Diagram showing a cross-section of a Phytostrip in place, seated on a 96-well microtitre plate. The Phytostrip is filled with nutrient gel and seed sown on the surface of the gel germinates and roots grow down through the gel where they can be imaged through the transparent walls of the Phytostrip. (Shoot development can also be followed by imaging from above). The Phytostrip is shown in contact with liquid medium contained in the wells of the microtitre plate. Chemical treatments can be applied by adding compounds of interest to the liquid medium and these are able to diffuse through the gel to be absorbed by the growing roots

The Phytostrips (Fig. 1a) were sealed at their bases with a 117 × 80 mm adhesive polyester film (PCR Seal, cat. no. 4ti-0500, 4titude Ltd, UK) to allow them to be filled with hot Phytagel medium. To do this, each set of 12 Phytostrips was assembled in a V-bottom 96-well microtitre plate (Anachem Ltd., UK, model no. ABAP-V) and locked together using a custom-made clamping device (see Additional file 1: Figure S1) which allowed the Phytostrips to be lifted from the microtitre plate as a block and inverted for the adhesive film to be applied (Fig. 1b). (A detailed engineering drawing of the clamping device and the other custom-made devices described below can be found in Additional file 2). Filling with hot Phytagel medium (ca 320 µl per well) was done either manually, using an 8-channel pipettor, or using a semi-automated liquid handling system (Viafill Dispenser, Integra Biosciences AG, Switzerland). Once the gel had set, the adhesive film was removed and the Phytostrips placed in a microtitre plate, each well of which contained 150–200 µl nutrient medium. Seed were sown on the gel surface either individually or (in the case of Arabidopsis only) as clusters of 5–7 seed per well using a 200 µl pipettor fitted with a cut-off tip to deliver droplets of a seed suspension. After sowing was complete, the assay plates were transferred to custom-made plate-holders and covered with a clear plastic box (Fig. 1c) before being moved to the growth room.

### Chemical treatments

Chemical treatments were applied after the seed had germinated and once roots were visible in the majority of the wells of the Phytostrips (3–4 days after transfer to the growth room). To initiate the treatment the Phytostrips were transferred to fresh 96-well plates containing 150–200 µl nutrient medium in each well and the desired concentration of the chemical being tested. For screening the 800 molecules from the Microsource Spectrum library the wells of the treatment plate contained 150 µl nutrient medium to which was added 1.5 µl of a 2.5 mM solution in dimethyl sulphoxide (DMSO) to give a theoretical final concentration of 8 µM of each small molecule (and 0.33% DMSO) after its diffusion into the gel. We observed no significant effect of the DMSO on root development under these conditions, but all seedlings in the screen were exposed to exactly the same DMSO concentration and the phenotypes elicited by our ‘hit’ compounds are those that are distinguishable from the >90% of the treatments that displayed the control phenotype.

### Histochemical β-glucuronidase (GUS) assay

The method used to histochemically stain roots of GUS reporter lines growing in the Phytostrips was based on that originally described [23], except that the roots were stained in situ by allowing the assay solution to diffuse into the gel in which they were growing. When the roots had grown to about two-thirds the depth of the Phytostrip (after 6–7 days of growth), staining was initiated by removing the nutrient solution from the microtitre plates and replacing it with 200 µl of a modified assay solution containing 2 mM 5-bromo-4-chloro-3-indolyl β-d-glucuronide (X-Gluc) in 0.25 M potassium phosphate buffer pH 7.0, 25 mM Na EDTA pH 7.0, 0.5 mM K ferricyanide, 0.5 mM K ferrocyanide and 0.25% (w/v) Triton X-100. The plates were then incubated in the dark at 29° for 24 h in a box containing moistened paper towel to maintain humidity, before being returned to the growth room for robotic imaging. To improve the visibility of the indigo-blue stain a white background was placed behind the Phytostrips when images were being captured.

## Results

### The ‘Phytostrip’: a novel plant growth device to enable automated imaging and analysis of root architecture in the context of a chemical screen

In the original version of the micro-phenotyping technique, commercially available strips of PCR tubes were filled with solid nutrient medium and their tips excised to allow chemical treatments to be applied by diffusion from the wells of a microtitre plate [17]. However, automated imaging was found to be impractical with these tubes because of reflections and distortions caused by their curved walls. Furthermore the conical shape of the tubes allowed roots to develop in three dimensions and to obscure one another, making quantitative analysis of root architecture unreliable. We therefore sought to develop an alternative version of the strips that would allow images of the roots to be captured easily and where the roots would grow in an essentially 2-D conformation. Growing roots in devices that oblige roots to develop in a 2-D space is a common practice in the study of root development because of the way that it simplifies the capture and analysis of root images [24, 25].

The design for the new device (the ‘Phytostrip’) is shown in Fig. 1a. It has eight growth vessels or wells that have flat sides and a rectangular cross-section with an internal distance from front to back of 2 mm and an overall height of 23 mm. The growth vessels are open at both ends and widen at the top to facilitate filling with gel (Phytagel) and seed sowing. The Phytostrips have been designed to fit a commercially available 96-well microtiter plate (Anachem model ABAP-V) and flanges at either end stabilise it when seated in the plate. A gap of 3 mm between the bottom of the Phytostrip wells and the bottom of the V-shaped wells of the microtitre plate allows roots to emerge from the Phytostrips unhindered. The Phytostrips are rigid to facilitate robotic handling and their wells are slightly tapered so that the device drops easily into the wells of the microtiter plate. For imaging purposes, and for chemical resistance, they are manufactured in clear medical-grade polypropylene.

The diagram in Fig. 1d illustrates how, in use, the Phytostrip sits within the wells of a 96-well microtitre plate, with the bottoms of the growth vessels immersed in 150–200 µl liquid medium. The liquid medium serves the dual purpose of providing a reservoir of nutrients and water (to counteract nutrient deficiency in the seedlings and desiccation of the gel) and a volume of liquid to which the test compounds can be added for diffusion into the gel. Sterilised seeds are sown onto the surface of the gel and the test compounds are normally added to the liquid medium on the first day that roots are visible (to avoid any potential effects on germination). Diffusion of the small molecules through the gel is relatively rapid (estimated at ≥0.8 mm h^−1^) as shown by the observation that, when a compound is particularly bioactive (such as a known plant hormone), it is possible to detect its effects in roots at the top of the growth vessel within 24 h of its application (data not shown).

It should be noted that, in common with most previous studies where seedlings are grown on vertical agar plates, the roots growing in the Phytostrips are exposed to ambient light from the overhead illumination. No significant shading of the roots occurs until the shoots start to develop, after which there will be a progressive reduction in the light intensity at the root surface. Owing to the physical constraints imposed on a system that allows whole seedlings to be imaged robotically at regular intervals and at visible wavelengths, it is not feasible to keep the roots in the dark. However, it should be recognized that light can permeate up to several millimeters in soil [26], and it can also be conducted several centimeters through the vascular system of the root [27], so that exposure of roots to some light is not necessarily unphysiological [28]. Although a recent comparison of light- and dark-grown Arabidopsis roots revealed differences in root growth and numbers of lateral root primordia, as well as some quantitative differences in sensitivity to hormones and abiotic stresses [29], these are not more significant than might be seen for example in roots growing under different nutrient conditions [30–32].

### The Microphenotron: a robotic platform for automated image capture from the Phytostrips

**Fig. 2:**
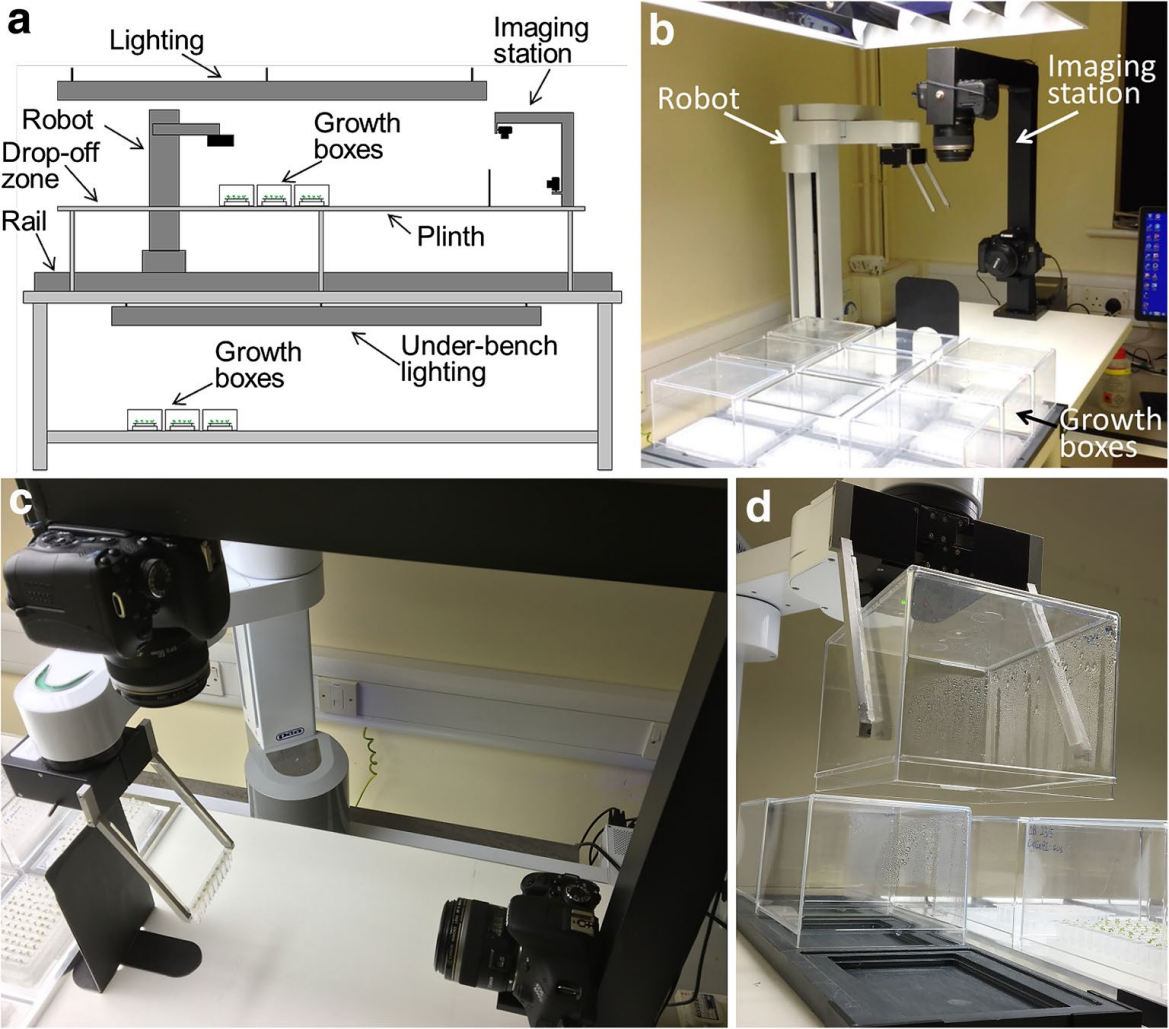
The robotic platform. **a** Line diagram showing the arrangement of the main elements of the Microphenotron. Note that a second illuminated shelf, with similar lighting and temperature conditions as the upper shelf, has been installed underneath the bench to double the number of assay plates that can be run at any one time. For imaging these, the assay plates on their plate-holders are moved to the upper shelf. The upper and lower shelves can hold up to three plate-holders, giving a capacity on each shelf of 27 assay plates in their growth boxes. **b** View of one end of the robotic platform showing a set of nine growth boxes in position, the robot in its resting position and the imaging station with its two cameras for imaging the Phytostrips and the side. **c** A close-up of the imaging station with a Phytostrip being presented by the robot for automated imaging by the two cameras, which are controlled by the same PC that operates the robot. The custom-made aluminium fingers that the robot uses to lift the Phytostrip can be clearly seen. **d** Image showing the dual-purpose fingers being used to remove a growth box before starting to collect individual Phytostrips for imaging. The robot is shown in the process of placing a box in the drop-off zone at the end of the plinth furthest from the imaging station

The general layout of the robotic platform is illustrated in Fig. 2. To reduce the cost and complexity of the system, the robotic platform has been designed to circumvent the need for computer vision to locate the objects to be moved (i.e. the Phytostrips) and a commercially available laboratory robot has been used (details below). The robotic platform comprises an illuminated bench that supports both the robot itself and a plinth on which up to 27 assay plates can be held in a fixed array (each assay plate comprising one set of 12 Phytostrips installed in a 96-well microtitre plate), allowing the robot to locate individual Phytostrips based only on their *x*, *y* coordinates (Fig. 2a, b). An additional illuminated shelf underneath the bench provides places for a further 27 assay plates (Fig. 2a). To maintain humidity, each assay plate is housed in an inverted clear polystyrene box of dimensions 141 × 117 × 95 mm (L × D × H) (Gard Plasticases Ltd, UK; cat no. 35159-010). The robot is equipped with custom-made fingers that enable it to pick up and move the plastic box to a drop-off zone at one end of the bench (Fig. 2d) and then to select each Phytostrip in turn and present it to an imaging station located at the other end of the bench. The imaging station consists of two cameras for capturing images from above and the side of the Phytostrip (Fig. 2c). After image capture the Phytostrip is returned to its original position and when imaging of one column of three plates is complete the robot replaces the plastic boxes (see video in Additional file 3). A complete set of 24 images from an assay plate can be captured in under 4 min, so that a whole shelf of 27 plates can be imaged in about 2 h, a rate equivalent to 2.5 s per individual well. With the additional capacity of the lower illuminated shelf, the Microphenotron platform has the capacity for up to 4320 individual assays to be performed simultaneously (80 per plate, allowing one strip at each end of the plate for controls) and robotically imaged each day to monitor both root and shoot development. Details of each component of the robotic platform are as follows:

#### The robot and its custom-made ‘fingers’

Robotic manipulations are performed by a Peak ProNEDx-450-500 laboratory robot (Peak Analysis & Automation Ltd, UK) mounted on a single-axis linear actuator rail (Fig. 2a-c). This robot has four degrees of freedom, including two rotational (shoulder and wrist) and two prismatic (arm and z) joints. When combined with the 2 m horizontal rail it is able to reach a cuboid volume of 2000 × 450 × 500 mm (length x depth x height) in front of it. The high-precision servomotors provide repeatability of ±0.08 mm, which is suitable for picking up the Phytostrips using open-loop control software (see Additional file 4). The robot is fitted with a servo-electric gripper to which has been fitted a pair of dual-function ‘fingers’ that enable the robot to pick up (in separate actions) both the plastic box that covers the assay plates and the individual Phytostrips (Fig. 2c, d). The gripper has a stroke of 54 mm and can close to a specified reaction force (100 N), enabling it to lift unknown objects whilst ensuring a tight grip. The rigid lightweight fingers were custom-made from 7075 aluminium alloy using computer numerical control (CNC) milling (Proto Labs Ltd, Telford, UK). Their angled design (see Additional file 1: Figure S3) was determined by imaging constraints, allowing both for an unobstructed view of the seedlings from the overhead camera and for the Phytostrips to be placed in front of a black background for imaging from the side. To provide a grip when lifting the boxes, a 2 mm thick silicone rubber insert was glued to the inside of each finger. The insert was made using ‘addition cure’ silicone rubber (Easy Composites Ltd, UK), cut to size with a craft knife after curing.

#### Supporting structure and lighting

The robotic platform is mounted on a free-standing heavy duty 2000 × 900 mm bench (Cubio Bench; Bott Ltd, Bude, UK). The 2 m linear-actuator rail (Peak Analysis & Automation Ltd, UK) is bolted onto the benchtop, allowing the ProNedx robot to traverse most of the length of the bench. To raise the assay plates to a height where they can be accessed by the robotic arm, a 2000 × 468 mm plinth with a height of 380 mm is fixed to the top of the bench, parallel with, and in front of, the linear-actuator rail (Fig. 2a). Illumination for plant growth is provided by a luminaire (Model Opti-Lux 2 T5 Low energy Low Bay, Ansell Lighting Ltd, Warrington, UK), dimensions 1490 × 413 × 105 mm (L × W × H), equipped with four Philips TLD 840 T5 80 W lamps and integrated ‘switchDIM’ digital dimming. A second identical luminaire fixed to the underside of the benchtop provides illumination for another shelf on which up to 27 further assay plates can be incubated and moved when necessary to the upper shelf for imaging. The distance between the luminaire and the assay plates was set up to be the same on the upper and lower shelves and the average light intensity on both shelves at plant height is 70 µmol m^−2^ s^−1^.

#### Plate-holders

The assay plates are arrayed on the plinth by placing them into custom-made portable plate-holders (389 × 461 × 12 mm) each holding up to nine plates in a 3 × 3 grid (Fig. 1c and Additional file 1: Figure S2). The plate-holders provide a way of fixing the precise *x*, *y* position of the assay plates on the platform, as required by the robotic handing system, and are also used as trays for transferring the assay plates between the flow bench and the growth room. The plate-holders were manufactured from aluminium tooling plate (alloy 5083) by AJ Engineering NW Ltd (Lancaster, UK). Up to three plate-holders can be arranged side-by-side on the plinth, secured by angle brackets. Within the plate-holders, the assay plates are held in position by being seated in an 85.75 × 128.05 mm indentation (Fig. 1c and Additional file 1: Figure S2) and the plastic boxes that cover the assay plates are seated on another, higher tier of indentation with dimensions 137.6 × 113.6 mm, the edges of the indentation being chamfered to help re-seat the box when it is returned to its location by the robot after imaging. A reservoir underneath the microtitre plate holds a moistened blotting pad (Bio-dot filter paper, Bio-Rad, UK) and is kept topped-up with water to maintain humidity inside the box. Grooves at either end of the indented area extend underneath the edge of the plastic box to allow for gaseous diffusion between the outside and inside of the box and to provide access for adding more water with a long-needled syringe when needed. An additional plate-holder with only three holding positions is located at the end of the bench furthest from the imaging station to provide a drop-off zone for the robot to place the plastic boxes while Phytostrips are being imaged (see Fig. 2a, d).

#### Imaging station

A simple and inexpensive framework for the imaging station was constructed by welding a pair of steel box sections to form an ‘L’ shape and adding two rectangular metal brackets to hold the cameras (Fig. 2b, c). Two digital cameras (Canon EOS 600D with Canon EF-S 60 mm f/2.8 macro USM lenses), one for imaging from above and the other from the side, are attached to the brackets via their tripod mounting holes using 19 mm long × 6.35 mm diameter Whitworth bolts. The cameras are positioned so that their lenses are each 225 mm from the part of Phytostrip being imaged, allowing the capture of overhead and side images of all 8 wells in the Phytostrip in each frame. The cameras are powered by mains adapters and connected to a central PC by USB cables. Using the software on the PC the cameras are set for 1/50th sec exposure time, f/2.8 aperture, ISO setting 200 and ‘large fine’ picture quality (18 megapixels). Image capture is activated by bespoke software (see Additional file 4), with images being downloaded directly to the PC as JPEG files which are automatically labelled to indicate the exact origin of the imaged Phytostrip on the platform and which source camera was used (e.g. PlateA4_Strip9_Side or PlateA4_Strip9_Top). The operator can use the interface software (see Additional file 4) to enter additional information that is common to all Phytostrips being imaged (e.g. the experiment number) and this will appear at the beginning of all filenames in that session.

**Fig. 3:**
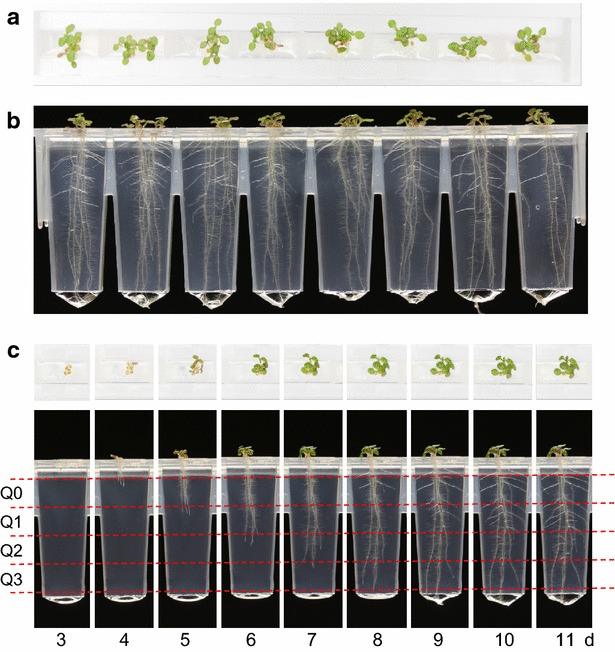
Robotically captured images of Arabidopsis seedlings growing in the Microphenotron. Clusters of 5–7 seed were sown on the surface of each gel-filled well of the Phytostrips and the assay plates incubated in the growth room under standard conditions (as described in "Methods"). **a** Image of one Phytostrip taken with the overhead camera showing shoots of seedlings 11 days after transfer to the growth room. **b** Image showing roots of the same seedlings as in **a** taken with the side camera. **c** A daily time series showing the stages of germination and growth of shoots and roots in a single well of the Phytostrip, starting 3 days after transfer to the growth room. The *red dotted lines* illustrate the quadrants into which the root images were divided as part of the automated image analysis (see Fig. 5 and associated text)

Indirect lighting for image capture is provided by the luminaire that illuminates the plants, avoiding the need for dedicated lighting. To provide contrast for optimal imaging of the roots, a matt black card is fixed vertically behind the Phytostrip when viewed from the side camera (Fig. 2b, c), while the white surface of the plinth provides a suitable background for the shoots when imaging from above. Examples of images of Arabidopsis seedlings growing in the Phytostrips and captured using the robotic platform, including a time series of images of roots and shoots captured on successive days, are shown in Fig. 3.

### Validating the Microphenotron

To demonstrate the capabilities of the Microphenotron we have considered two contrasting scenarios in which it may be exploited. The first is where the aim is to screen a large number of different molecules for their effect on one or more aspects of the plant phenotype (i.e. a typical chemical genetics screen). In this case the number of replicates will necessarily be small (normally two), so to avoid a large number of false positives it is essential that there is a very clear and reproducible difference between the sought-after phenotype (the one that will be scored as a ‘hit’) and the normal range of background phenotypes. The second case is where the aim is to screen a much smaller number of samples (perhaps at a range of dilutions) but to combine this with a large number of replicates (16 or 24, corresponding to two or three Phytostrips), making it possible to detect relatively small effects on root or shoot growth. In the former case, an effective primary screen could be achieved by simple visual inspection of the images, as was successfully done when the manual version of the microphenotyping technology was used to identify small molecule antagonists of glutamate’s effect on root architecture [17]. In the latter case, however, it will be necessary to extract quantitative data from the images to identify subtle effects, and because of the very large numbers of images potentially involved, it is essential that this can be done with minimal human intervention using purpose-built software. Experimental validation of the Microphenotron in these two scenarios is described below:

#### A screen of 800 natural products identifies a set of molecules eliciting distinctive root phenotypes

**Fig. 4:**
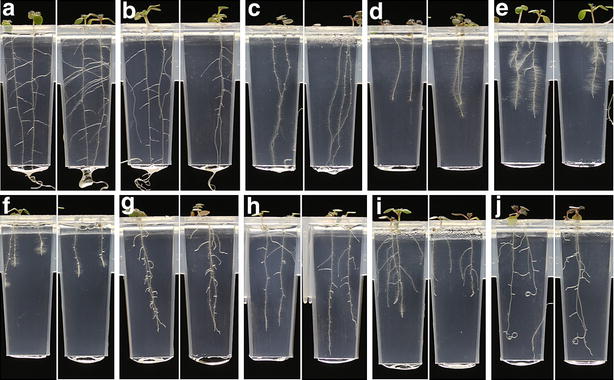
Using the Microphenotron to screen a set of 800 molecules for effects on root system development. Sets of twelve Phytostrips filled with nutrient gel were set up in 96-well microtitre plates containing 150 µl nutrient solution per well. Arabidopsis seed was sown on the gel surface (2 seed per well) and the assay plates were placed in the plate-holders, covered with the growth boxes and transferred to the robotic platform. After 3 days, when most roots were first visible, the Phytostrips were transferred to fresh microtitre plates containing chemicals from the compound library at a starting concentration in the microtitre plate of 25 µM (final theoretical concentration after diffusion = 8.3 µM) and returned to the robotic platform. Each set of 80 chemicals from the library was assayed in duplicate plates and images from the 20 plates were captured 5–8 days after treatment and scored visually for altered root phenotypes that were seen in both replicates. The images shown were selected as representative of the range of root phenotypes observed. The treatments and the number of days after treatment (d.a.t.) that the images were captured were: **a** control (7 d.a.t.); **b** deoxycholic acid (8 d.a.t.); **c** abscisic acid (8 d.a.t.); **d** 10-hydroxycamptothecin (7 d.a.t.); **e** aminocyclopropanecarboxylic acid (7 d.a.t.); **f** sodium phenylbutyrate (5 d.a.t.); **g** 5α-androstanedione (7 d.a.t.); **h** 5,7,4′-trimethoxyflavone (5 d.a.t.); **i** ethionine (5 d.a.t.); **j** diffractaic acid (8 d.a.t.)

The Microsource NatProd Collection (Microsource Discoveries Inc., USA) comprises a set of 800 natural products and their derivatives, originating from plant, animal and microbial sources. Only 41% of these compounds are listed as having known biological activity, and of these only eight as having known activity in plants. We have used the Microphenotron to screen these 800 molecules in duplicate for their effects on Arabidopsis root growth. Seed was germinated and grown in the Phytostrips for 3 days (to give time for roots to appear) and the chemicals were then added to the nutrient solution in the microtitre plates so that they reached the growing roots by diffusion through the gel (see Fig. 1d). This allowed effects on seedling development to be observed independently of any potential effects on germination. Images captured robotically 5–8 days after treatment were examined visually for significant alterations to the root phenotype that were consistent across the replicate treatments. Since two seed were sown per well, in most cases there were at least four individual root systems in which to observe alterations in the phenotype. A representative selection of the range of root phenotypes observed and their reproducibility in replicate treatments is shown in Fig. 4. It was possible to visually score nine root traits from the captured images: (1) inhibition of primary growth; (2) increase/decrease in lateral root density; (3) inhibition of lateral root growth; (4) longer/shorter root hairs; (5) presence/absence of root hairs; (6) distribution of root hairs along the root; (7) agravitropism/wavy growth (of primary and/or lateral roots); (8) root tip morphology; (9) root thickening. The effectiveness of the morphological screen was based on the human brain’s innate expertise at pattern recognition, currently superior to machine learning approaches [33].

**Fig. 5:**
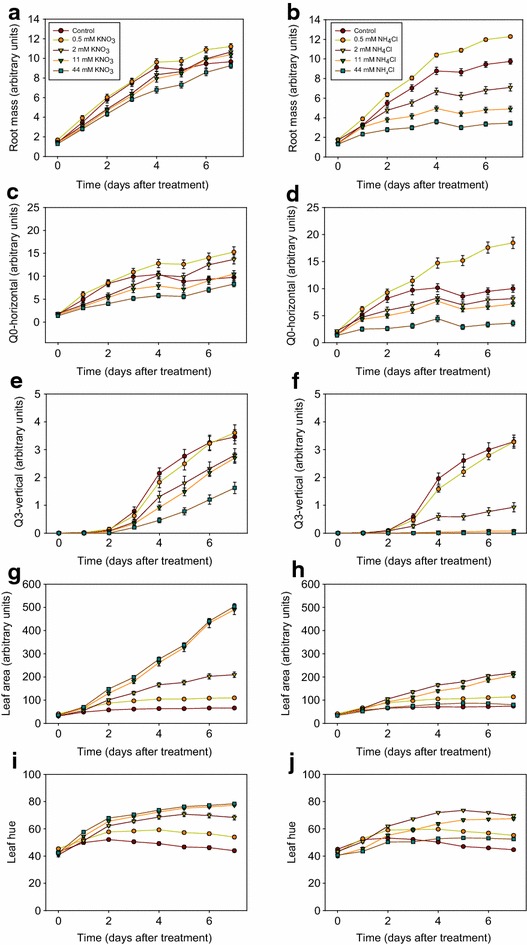
Time courses showing the effects of a range of KNO_3_ and NH_4_Cl concentrations on Arabidopsis root and shoot development. Images were captured robotically by the Microphenotron and quantitative data generated using fully automated image analysis software (AutoRoot). A suspension of Arabidopsis seed that had been surface-sterilised and soaked in water at 4^o^ for 48 h was sown onto the gel surface of the Phytostrips as clusters of 5–6 seed per well. The initial N supply in the gel of the Phytostrips and in the 150 µl liquid medium in the microtitre plates was 0.05 mM KNO_3_. After 4 days in the growth room, when most roots were first visible, the nutrient solution in the microtitre wells was replaced with a fresh nutrient solution containing the appropriate concentration of either KNO_3_ or NH_4_Cl to give a final theoretical concentration of 0.5, 2, 11 or 44 mM of the respective N source after diffusion into the gel. The control treatments contained only 0.05 mM KNO_3_. Three replica 96-well plates were set up, each with a full set of all eight N treatments (one per Phytostrip) plus two control Phytostrips (the Phytostrips at either end serving as buffers against edge effects). Images of the developing seedlings were captured robotically from above and the side on the day of treatment and daily thereafter and analysed using the AutoRoot software [19]. Time-courses of the development of five of the software-generated traits have been plotted: **a, b** ‘root mass’, a likelihood estimate of the total root mass in each well; **c, d ‘**Q0-horizontal’, a trait that allows lateral root branching in the top quadrant of each well to be tracked; **e, f** ‘Q3-vertical’, a trait that allows the extent of primary root growth in the bottom quadrant of each well to be tracked; **g, h** ‘leaf area’, a measure of the total area of leaves as viewed from above (an underestimate of the actual leaf area at later time points because of overlap between leaves); **i, j** ‘leaf hue’, a measure of the greenness of the leaves (n = 24 ± SE). The scale for hue on the *y* axis is from 0 (*red*) to 120 (*green*)

This analysis revealed that 70 of the 800 molecules in the compound library elicited a clearly modified root phenotype (an 8.8% hit rate). Some phytoactive molecules may have been missed because of occasional loss of one or both replicates (e.g. due to microbial contamination or failure of germination). Thirteen of the compounds caused loss of root hairs, and for 8 of these compounds this was the only observed effect (e.g. deoxycholic acid, Fig. 4b). Twenty four compounds partially or completely inhibited lateral root development while having little or no effect on primary root development at the concentration used. One of the molecules in this group was abscisic acid (ABA; Fig. 4c), consistent with previous studies showing that early lateral development is particularly sensitive to an external ABA treatment [34]. Another group of 16 compounds strongly inhibited both primary and lateral root growth (e.g. 10-hydroxycamptothecin, Fig. 4d). Aminocyclopropanecarboxylic acid (ACC), a precursor of the plant hormone ethylene, inhibited both primary and lateral root growth and strongly stimulated root hair elongation (Fig. 4e), which is the same phenotype previously reported for roots treated with exogenous ACC [35, 36]. Roots were also highly sensitive to inhibition by sodium phenylbutyrate (Fig. 4f), a histone deacetylase inhibitor and chemical chaperone [37] that has not previously been reported to have major effects on root development. 5α-androstanedione was one of four steroid hormones in the library to affect root development, particularly lateral root development (Fig. 4g), and 5,7,4′-trimethoxyflavone was one of seven flavones or isoflavones (out of 32 in the library) to which root development was sensitive in one way or another (Fig. 4h). One molecule (ethionine) was unusual in strongly inhibiting primary root growth while having little effect on lateral root growth (Fig. 4i) and four compounds induced some form of agravitropic or wavy root growth (e.g. diffractaic acid, Fig. 4j).

This experiment illustrates the sensitivity of root development to low concentrations of diverse chemical compounds and the variety of root phenotypes that can be detected using the Microphenotron technology. Furthermore in cases where the hit compounds had known bioactivity in plants (i.e. ABA and ACC), the phenotypes obtained were fully consistent with those previously reported. Although no auxin-related molecules were present in the library, we have shown in a separate experiment (see Additional file 1: Figure S4) that indole acetic acid (IAA) has the expected effect of inhibiting primary root growth and stimulating root hair elongation [35, 38].

#### Quantitative analysis of the effect of a range of concentrations of nitrate and ammonium on root and shoot development

To demonstrate the ability of the Microphenotron to detect more subtle quantitative effects on seedling development, we performed an experiment in which seedlings were treated with a range of concentrations of KNO_3_ and NH_4_Cl. Differences from the previous experiment are that 5–6 seed were sown as a cluster in each well and three Phytostrips were used for each treatment, giving 24 replicates and a total of over 100 individual seedlings per treatment. It was found in preliminary experiments that the larger number of seedlings per well was important for obtaining the level of reproducibility between wells needed to detect smaller-scale effects. In this experiment, seed was germinated on standard medium containing only 0.05 mM KNO_3_ as N source. Treatments with a range of concentrations of KNO_3_ or NH_4_Cl (0–44 mM) were initiated 4 days after sowing, when roots were first visible, and images were captured on the day of treatment (0 d.a.t.) and then daily up to 7 d.a.t.

Because of the large number of images of individual Phytostrip wells that are generated in a typical Microphenotron experiment (in this case 24 side and 24 overhead images per treatment per day, giving a total of 3840 images over 7 days), to avoid a bottleneck at the data-processing stage it was necessary to develop novel software that would allow fully automated extraction of quantitative data from these images. The accompanying paper [19], describes software (AutoRoot) which uses an alternative probabilistic-style approach to quantifying root growth inside each well. AutoRoot allows images to be analysed batch-wise without human intervention at a rate of about 4 s per Phytostrip well. At the same time, AutoRoot also generates data quantifying shoot development from the overhead images.

**Fig. 6:**
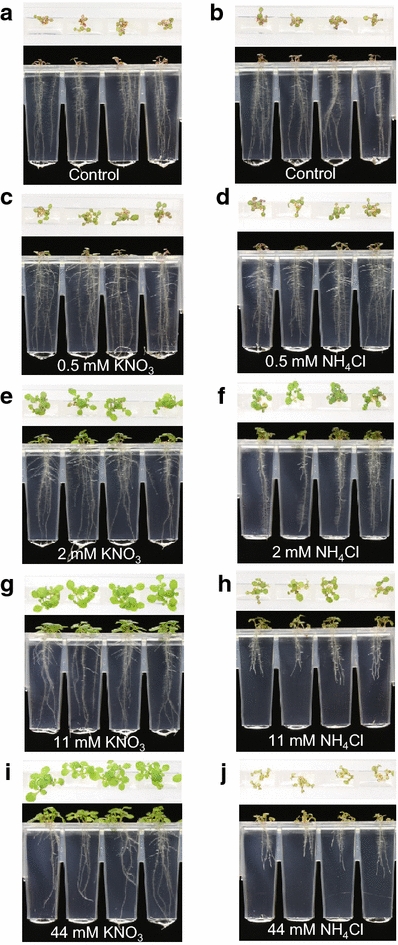
Images showing the effects of a range of KNO_3_ and NH_4_Cl concentrations on Arabidopsis root and shoot development. The images, captured from above and the side at the 5 d.a.t. time point, are a representative set from the experiment described in Fig. 5. The left-hand set of images show the NO_3_^−^ treatments and the right-hand set the NH_4_^+^ treatments: **a, b** control; **c, d** 0.5 mM; **e, f** 2 mM; **g, h** 11 mM; **i, j** 44 mM

AutoRoot was used to analyse the images captured in this experiment and five of the traits extracted by the software from each well were selected for presentation in Fig. 5: ‘root mass’, which is a likelihood estimate of the total number of root pixels; ‘Q0-horizontal’, which is an estimate of the number of pixels in the top quadrant (see Fig. 3b) that correspond to root material with an orientation between 0° and 22.5° from the horizontal (mainly corresponding to root hairs and lateral roots); ‘Q3-vertical’, which is an estimate of the number of pixels in the bottom quadrant that correspond to root material with an orientation between 67.5° and 90° from the horizontal (corresponding to primary roots, until the lateral roots from the upper zones eventually extend into this quadrant); ‘leaf area’, which is based on a count of all coloured pixels in the image captured from above, and ‘leaf hue’, which is a measure of the colour of the shoots on the red-green scale (where red = 0 and green = 120). Figure 5 shows the time-course of development of each of the five traits, and for comparison Fig. 6 shows a representative set of images from the 5 d.a.t. time point. For full details of all the parameters that are generated by AutoRoot see the accompanying paper [19].

The ‘root mass’ data (Fig. 5a, b) show the different ways in which KNO_3_ and NH_4_Cl affect the development of the Arabidopsis root system over time and are consistent with what is seen in the images (Fig. 6). Compared to the controls (0.05 mM KNO_3_), the supply of 0.5 mM additional N (as NO_3_^−^ or NH_4_^+^) stimulated root growth, although in the case of KNO_3_ this was solely by prolonging the period of root growth beyond 4 d.a.t. when growth ceased in the controls (presumably due to exhaustion of the small amount of N in these wells). Higher concentrations of KNO_3_ were slightly inhibitory compared to 0.5 mM KNO_3_, whereas concentrations of NH_4_Cl of 2 mM and above were strongly and increasingly inhibitory. This is consistent with many previous reports of the inhibitory effects of high NH_4_^+^ concentrations on root growth when NH_4_^+^ is supplied as sole N source [39]. The ‘Q0-horizontal data’ show a stimulation at 0.5 mM of both N treatments that becomes apparent from 4 d.a.t. (Fig. 5c, d) and corresponds to the stage when lateral roots begin to appear (equivalent to 8 days after sowing in the time course shown in Fig. 3b). That this reflects a stimulation of lateral root development is confirmed by inspection of the images in Fig. 6 (cf. Fig. 6a, b with Fig. 6c, d), supporting the use of this trait as a proxy for detecting effects on root branching. The ‘Q0-horizontal’ data also show that as the concentrations of both N sources, particularly NH_4_Cl, were raised they had increasingly negative effects on lateral root development compared to the 0.5 mM treatments (Fig. 5c, d). The ‘Q3-vertical data’ (Fig. 5e, f), which report the amount of downward growing root material in the bottom quadrant, are consistent with the effects on primary root growth that can be seen in the images in Fig. 6. The ‘Q3-vertical’ data show that the highest concentration of KNO_3_ (44 mM) had a strong negative effect on primary root elongation (Fig. 5e) and that NH_4_Cl concentrations of 2 mM and above were also strongly inhibitory. The causes of the negative effects of NH_4_^+^ on root growth are known to be complex and may at least in part be due to acidification of the rhizosphere [39], but we were not concerned here with the underlying mechanisms only in demonstrating the ability of the technology to quantify diverse changes in the root phenotype.

The two parameters that were used to monitor shoot development, leaf area and leaf hue, correlated well in their responses to the two N sources (Fig. 5g–j) and matched what could be seen in the images taken from above (Fig. 6). In the case of KNO_3_, increasing concentrations in the range 0.5–11 mM led to both a stimulation of leaf expansion (Fig. 5g) and an increase in leaf hue (i.e. greenness, Fig. 5i), with a further increase to 44 mM having no additional effect on either trait. We interpret the gradual decline in leaf hue seen at the two lowest KNO_3_ concentrations as a sensitive indicator of the onset of seedling stress, in this instance presumably due to N deficiency. In the case of NH_4_Cl, the optimum concentrations for both leaf expansion and leaf hue were between 2 and 11 mM (Figs. 5h, j, 6f, h), although the positive effects on leaf expansion were much weaker than those seen for KNO_3_ (Figs. 5g, 6). There is an interesting contrast here with the effects on root development, which were strongly negative at the concentrations of NH_4_Cl that were optimal for shoot growth (Figs. 5b, d, f, 6f, h).

These results demonstrate that the Microphenotron platform, when combined with automated image analysis software (AutoRoot) and sufficiently replicated treatments, is able to generate a detailed and informative dataset that reports dynamic changes in both root system architecture and shoot development as seedlings respond to chemical treatments.

#### In situ histochemical assay for β-glucuronidase (GUS) reporter gene expression in Arabidopsis roots performed using the Microphenotron

The simplicity of the histochemical GUS assay, which involves formation of a blue precipitate by cleavage of the 5-bromo-4-chloro-3-indolyl-β-d-glucuronide (X-Gluc) substrate [23], and the availability of a wide variety of GUS reporter lines of Arabidopsis, make it a particularly attractive system for monitoring gene expression. It is therefore not surprising that the histochemical GUS assay has been used as the basis for a significant number of successful chemical genetic screens, leading to the identification of molecules that perturb the defence response [12, 40–42] and hormone signalling pathways [43–45]. With the aim of combining the convenience of the histochemical GUS assay with the other advantages of the Microphenotron, we decided to develop a protocol that would allow Arabidopsis roots in the Phytostrips to be stained for GUS activity while still located within the gel.

**Fig. 7:**
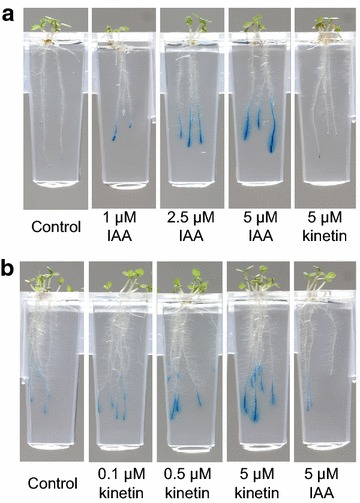
Histochemical stating for GUS expression in three transgenic reporter lines. Seed of each line (*DR5::GUS* and *ARR5::GUS*) was germinated and grown in the Phytostrips for 6–7 days. Hormone treatments were applied by adding concentrated stock solutions of **a** IAA or **b** kinetin to the nutrient medium in the microtitre plates and the plates were returned to the growth room for 24 h. To initiate staining for GUS activity in the roots, the nutrient medium in the microtitre plates was simply replaced with 200 µl assay solution containing X-Gluc (see "Methods" section). The Phytostrips were then incubated in the dark at 29^o^ for 24 h to allow time for the components of the assay solution to diffuse into the gel and for staining of the roots to develop. Images of the roots were captured using the robotic imaging system

Figure 7a shows the results obtained with the *DR5::GUS* auxin-responsive reporter line [20], which had been treated for 24 h with a range of concentrations of IAA before staining. Roots of the IAA-treated seedlings were histochemically stained by simply replacing the nutrient solution in the microtitre plate wells with an optimised assay buffer and allowing the reagents to diffuse into the gel in which the roots were growing. In the absence of added IAA, a small amount of localised staining could be seen in the root tips, corresponding to the ‘auxin maximum’, the accumulation of endogenous auxin that occurs in the vicinity of the columella initial cells [46]. In addition, as expected from previous studies (e.g. [47]), a number of lateral root primordia were stained, but only weakly (perhaps because of the relatively low nitrate concentration in the medium [48]) and the resolution of the image as reproduced in Fig. 7a does not allow these blue spots to be seen. As the concentration of exogenous IAA was increased, a clear increase in the intensity of blue staining in the primary root tips was evident, along with an increase in the extent of staining along the root. Similar results were obtained when the *ARR5::GUS* cytokinin reporter line [21] was pre-treated with a range of concentrations of kinetin before staining (Fig. 7b), although here the amount of staining in the untreated roots was higher than in those of the untreated *DR5::GUS* line. The specificity of the reporter lines for their respective hormones can be seen from the absence of any detectable effect of a 24 h treatment with 5 µM kinetin on *DR5::GUS* expression (Fig. 7a) and a similar lack of a detectable effect of a 24 h treatment with 5 µM IAA on *ARR5::GUS* expression (Fig. 7b). Leakage of blue stain into the surrounding medium was seen at the highest hormone concentrations, indicating that if more detailed information about the spatial localization of GUS expression were needed, shorter incubation times (and/or lower incubation temperatures) may sometimes be required. Note that since there is likely to be a vertical gradient in the concentration of the histochemical reagents as they diffuse through the gel, it is advisable to make direct comparisons of staining intensity only between roots of similar length.

### Growth of other plant species

**Fig. 8:**
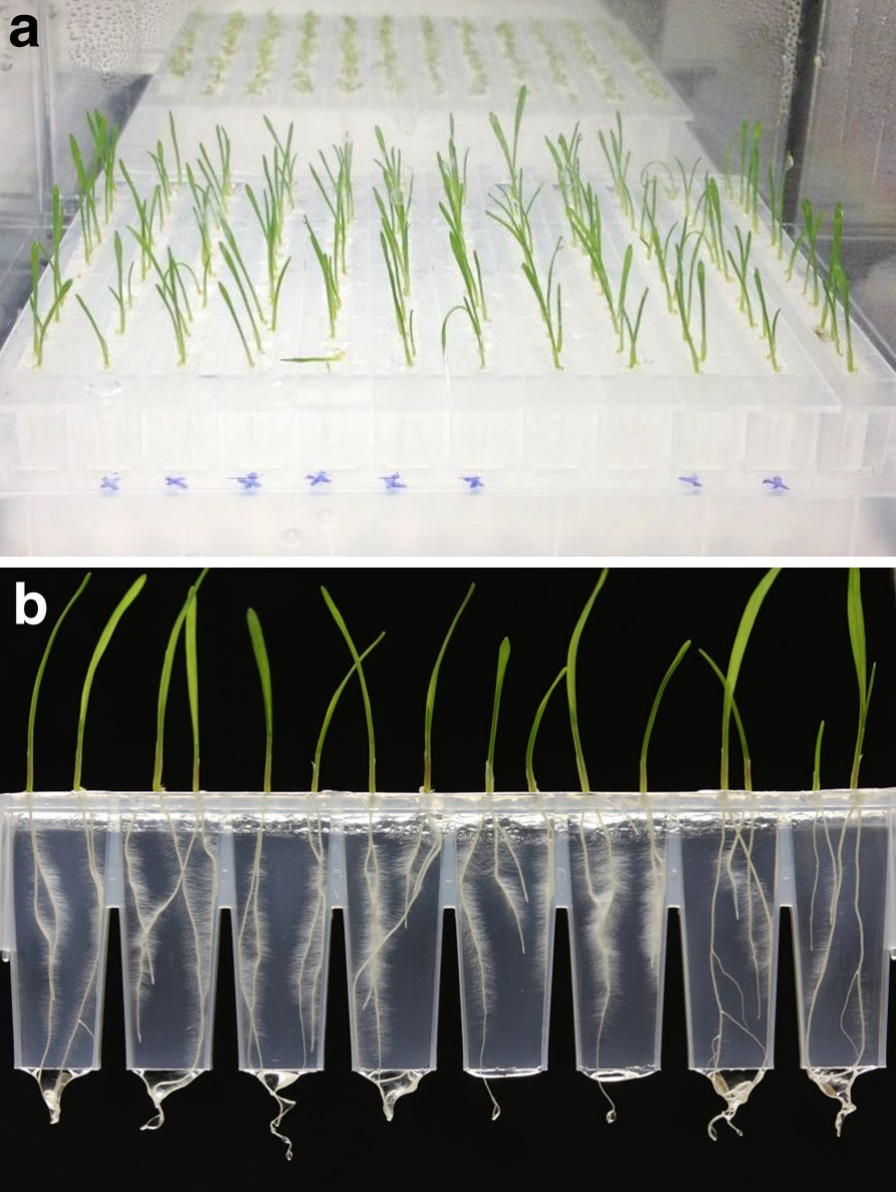
Growth of a monocot species, *E. tef* (teff), in the Microphenotron. Two teff seed (which had been surface-sterilised and kept at 4° for 48 h) were sown on the surface of each gel-filled well of the Phytostrips and cultured under the same conditions as used for Arabidopsis. **a** Image showing a whole plate of teff seedlings taken 5 days after sowing. **b** Robotically captured image showing 8 d-old teff seedlings growing in a Phytostrip

Arabidopsis is the species of choice for most chemical genetics applications, and it has been demonstrated that plant growth regulators identified from Arabidopsis screens are generally also active in a range of different crop species [4]. Although the Microphenotron has been designed for use with Arabidopsis, there is understandable interest in the possibility of cultivating other plant species in the system, particularly crop species. When testing a range of different species (including a number of commercial varieties of tomato and lettuce) it was found that early root development in most crop species is too vigorous for them to be suitable for growth in the current version of the Phytostrip. However, successful cultivation of the small-seeded cereal *E. tef* (teff), a major food grain in Ethiopia and Eritrea, was achieved. Figure 8 shows images of the teff genotype Tsedey, growing in the Phytostrips. The Tsedey genotype was chosen because it has been the subject of genome and transcriptome sequencing projects [49]. It was found that teff could be cultivated for 8 days or more under these conditions, giving time for the development of a first and second seminal root and the appearance in some individuals of the first lateral roots (Fig. 8b). It is likely that the Microphenotron will be suitable for studies on other plant species, the main requirements being small seeds (ideally <2 mm), good germination rates (high percentage and synchronous) and a not overly vigorous root system.

## Discussion

We describe a novel robotic facility that makes it possible to conduct high-content, miniaturized screens for the effects of small molecules on both root and shoot development in a 96-well microtitre plate format. At the heart of this automated platform is a novel seedling growth device, the Phytostrip, which has been specifically designed to allow detailed analysis of the effects of chemical treatments on root system architecture. Roots are a particularly attractive subject for phenotyping studies because of the large number of individual traits that can be readily visualised (Fig. 4) and the extent to which each of these traits is responsive to environmental factors [30, 50]. Many previous investigations into the genetic control of plant responses to abiotic and biotic stresses have focussed on root development [51, 52] and powerful robotic and imaging technologies have been developed to streamline the quantitative analysis of root growth and architecture of soil-grown roots [53–55]. However, combining large numbers of chemical treatments with detailed observations of root system architecture and root morphology has proved challenging. Previous chemical genetic approaches where root growth or morphology have constituted all or part of the readout have been relatively few [4, 14, 17, 56–58] and in most cases have focused on simple traits like root length, root tip swelling or gravitropic bending. Exceptions have been a screen for changes in root architecture [17], which used a non-automated precursor of the microphenotyping system described here, and a recent semi-automated screen for novel plant growth regulators in which root development was monitored by growing seedlings on the surface of solid medium in vertically-orientated 24-well plates [4]. Because the latter technology (which we will refer to as the ‘automated 24-well system’) currently comes closest to matching the specifications of the Microphenotron it is valuable to directly compare the operation and capabilities of the two systems.

In the ‘automated 24-well system’, the 15 mm diameter of the wells in the assay plate restricts primary root growth in the vertically orientated plates to ~10 mm, so that root imaging is necessarily limited to very young seedlings in which lateral roots are only just beginning to emerge [4]. In the Microphenotron, primary roots can grow to ~23 mm before emerging from the bottom of the Phytostrips, and thereafter they can continue growing for several more days in the nutrient solution contained in the microtitre plate wells. Thus root development can be followed for much longer, allowing development of a fully branched root system (see Fig. 3). In the ‘automated 24-well system’ (and in any screen where vertically orientated microtitre plates are used), the shoots are in direct contact with the surface of the nutrient agar, which is reported to cause artefacts in lateral root development due to sucrose uptake through the leaves [59]. In the Microphenotron, the shoots grow in the air above the surface of the gel, avoiding this issue. Furthermore, in the automated 24-well system (as in all previous agar-based systems) it is required that the chemical treatment is present in the agar at the time of germination. In the Microphenotron, by contrast, the chemical treatment can, if required, be delayed until after germination, so that effects on later developmental processes can be studied independently of any effects on germination itself. In terms of throughput, the ‘automated 24-well system’ uses an automated microscope to capture images of the seedlings at a rate of 50 s/well [4], compared to an imaging rate equivalent to 2.5 s/well in the Microphenotron. Finally, while we have developed tailor-made software for automated analysis of multiple root and shoot parameters in the Phytostrips [19], in the ‘automated 24-well system’ root length and leaf area were measured manually using the Root Tools macro in ImageJ [4].

The capabilities of the Microphenotron platform are also quite distinct from those of a number of specialized microfluidics-based systems that are designed for short-term fluorescence studies of much smaller numbers of roots using confocal microscopy [60, 61]. Another microfluidics set-up has been designed to allow longer-term studies of whole Arabidopsis seedling development [62], but in this case the small size of the growth chamber severely restricts the development of the root system both laterally and vertically, precluding detailed analysis of root architecture.

The optical resolution of the imaging system we have used for the Microphenotron is sufficient to allow us to detect individual Arabidopsis root hairs (diameter ~10 µM) in images that capture the entire width of a Phytostrip (e.g. see Figs. 3, 4), which satisfies our requirements to monitor external changes in root morphology. To obtain higher magnification for specific applications the cameras could be moved closer to the Phytostrips (the minimum working distance of the Canon EF-S 60 mm f/2.8 macro lens is 90 mm), requiring multiple images to capture each Phytostrip. For even higher degrees of magnification, and if throughput was not an issue, the Phytostrips can be removed individually and placed under a suitable microscope (see below).

Reporter genes provide a powerful tool for chemical genetic approaches and a recent survey found that of 40 reported chemical screenings in plants, 15 used transgenic Arabidopsis lines expressing either GUS, green fluorescent protein (GFP) or luciferase [63]. To expand the capabilities of the Microphenotron we have therefore developed a modification of the histochemical GUS assay to allow roots to be stained in situ with X-Gluc (Fig. 7). An auxin reporter line (*DR5::GUS*) and a cytokinin reporter line (*ARR5::GUS*) were used to demonstrate the ability of this technique, in combination with the appropriate reporter lines, to provide an assay for the presence of a range of different phytoactive molecules. The histochemical GUS assay described here has several advantages over methods previously used to stain roots for GUS activity. Firstly, the replacement of nutrient solution in the microtitre wells with the X-Gluc solution is simple to perform and the stained roots are readily imaged in the Microphenotron using the same automated image capture process that is used to follow root growth. Secondly, the stained roots are displayed in their original conformation, allowing the intensity of staining and its spatial distribution to be observed while retaining the information about root architecture that is a feature of the Phytostrip. Thirdly, no physical manipulation or movement of the roots themselves is involved, since replacement of the nutrient solution with the staining solution takes place in the wells of the microtitre plates, with the roots remaining embedded in the gel of the Phytostrips throughout. This avoids any possibility of damage to the fragile roots that can occur when they are immersed in a liquid medium that has to be removed and replaced with staining solution. Physical damage to the root may cause artefactual patterns of staining due to uneven penetration of the substrate into the tissue.

In addition to the reporter lines used in this study, the literature includes a variety of examples of GUS reporter genes that are expressed in roots and that are responsive to other phytohormones [64–67] or environmental factors [68–70], or which report on mitotic activity in the root meristem [71]. Although lines expressing marker proteins other than GUS have not yet been tested in the Microphenotron itself, GFP expression in root tips was previously detected in gel-filled Framestrips similar to the Phytostrips described here [17]. This was achieved simply by placing the Framestrips on the stage of a suitable fluorescence microscope, something that could equally be done with the Phytostrips.

Although the current version of the Microphenotron is set up to image only at visible wavelengths, it is possible to envisage imaging at other wavelengths that could provide more information on the physiological condition of the seedlings, as is frequently done in other phenotyping systems [52]. This could include chlorophyll fluorescence imaging (to monitor photosynthetic status and general leaf health) and thermal infrared imaging (to monitor leaf temperature as an indicator of stomatal conductance and water stress) [72].

The Microphenotron was primarily designed for applying chemical treatments through the roots, arising from our interest in using small molecules to manipulate biological processes in the roots. However, even for whole-seedling studies this method of application has a number of advantages over the alternative of applying the chemicals to the shoots. Firstly, uptake of the small molecules is favoured because roots have evolved for the purpose of absorbing chemicals (nutrients) from the soil and they also lack the waxy cuticle that can restrict the absorption of compounds through the leaf surface. Secondly, the gel in which the roots are growing, and into which the chemicals diffuse, allows prolonged contact of a large reservoir of the chemical with the root surface, something more difficult to achieve by spraying leaves. Thirdly, if mobility of the compound through the phloem is limited owing to its chemical properties, it can still have a systemic effect by being translocated through the xylem to the shoot in the transpiration stream. Finally, it is technically much more straightforward to apply chemicals individually to each well of the microtitre plate from below, than to achieve the same result by spraying from above. Nevertheless, if it is desirable to test the effect of foliar treatments, sprays could be applied to whole plates or to individual Phytostrips (after removal to a separate microtitre plate). However, because there is a delay of 1–2 days after roots are first visible before there is sufficient cotyledon/leaf area to spray, this reduces the number of days over which the effects on seedling development can be monitored.

Finally, although robotic handling of the Phytostrips for image capture is an essential part of creating an automated microphenotyping platform capable of imaging large numbers of seedlings on a daily basis, the robot is also by far the most expensive component of the Microphenotron facility, and much of the complexity in the design of the platform (i.e. the robotic plinth and the plate-holders) is driven by the robot’s requirement for the *x*, *y* coordinates of the Phytostrips to be precisely determined. Therefore, if throughput is not a major consideration, a laboratory could inexpensively assemble a simplified version of this micro-phenotyping system in a conventional growth room using only the assay plates (i.e. the Phytostrips + microtitre plates), clear plastic boxes to maintain humidity and a fixed pair of digital cameras to which individual Phytostrips could be moved manually for imaging from above and the side. AutoRoot, the software for automated analysis of the images [19], is Open Source and can be downloaded from https://zenodo.org/, and the Phytostrips are available to purchase by contacting the corresponding author.

## Conclusions

We have described the development of the Microphenotron platform as a novel and versatile addition to the array of plant phenotyping facilities already available [25, 73]. The specific advantages that the Microphenotron offers are: (a) the ability to follow both shoot growth and the development of root architecture under conditions where large numbers of chemical treatments can be applied; (b) the ability to allow the chemical treatments to be applied after germination and at several growth stages without disturbing the plants; (c) the ability to non-destructively image large numbers of seedlings rapidly and at daily intervals during the first 10–12 days of development; (d) the ability, in combination with purpose-built the AutoRoot automated image analysis software, to generate large amounts of quantitative data describing dynamic changes in root and shoot development; (e) the ability to perform these functions in small volumes (economising on chemical inputs), in a highly space-efficient way and with a minimal amount of manual input. Although primarily designed for chemical genetic applications the Microphenotron could also find applications in comparing the development of root architecture in large numbers of different Arabidopsis genotypes (e.g. for quantitative trait analysis or association mapping). Furthermore, without the need for the robotic component it could also be a valuable undergraduate teaching aid, a single assay plate with 12 Phytostrips providing ample scope for students to design a set of controlled and replicated experiments to investigate the effects of chemical treatments or environmental stresses on seedling development.

Because of the need to combine an automated screen with the application of large numbers of chemicals in small volumes under aseptic conditions, compromises have inevitably been required. Limitations of the system include the requirement to use sterile, transparent medium rather than soil, the relatively short length of time that seedlings can be grown (precluding studies of effects on flowering), and restrictions to the variety of species that can be cultivated. These limitations could mostly be addressed by developing a modified and scaled-up version of the Phytostrips, but the larger volumes of growth medium then required would only be acceptable when the experimental design was not limited by the cost and availability of the small molecules to be tested (e.g. in the case of biostimulants).

## Additional files

**Additional file 1.** **Figure S1.** The assembled clamping device used to hold a set of 12 Phytostrips in place so that they can be inverted and sealed at their base with adhesive film. **Figure S2**. 3-D drawing showing a cross-section of one of the plate-holders**. Figure S3.** Custom-made fingers for the robotic gripper. **Figure S4.** Images showing the effect of a range of IAA concentrations on root and shoot development and the reproducibility of the seedling phenotypes across each Phytostrip.

**Additional file 2.** Engineering drawings of the clamping device, robotic fingers and plate-holder.

**Additional file 3**. Video showing the Phytostrips and the Microphenotron in operation.

**Additional file 4**. A description of the custom control software for the robotic manipulator.

## Abbreviations

d.a.t.: days after treatment
GFP: green fluorescent protein
GUI: graphical user interface
GUS: β-glucuronidase
IAA: indole acetic acid
X-Gluc: 5-bromo-4-chloro-3-indolyl-β-d-glucuronide
