# Supplementary material for: The Microphenotron: a robotic miniaturized plant phenotyping platform with diverse applications in chemical biology
Source: Plant Methods. 2017 Mar 1;13:10. doi: 10.1186/s13007-017-0158-6 (PMC5333401; doi:10.1186/s13007-017-0158-6)
Supplement: Supplementary file 1 — Additional file 1. Figure S1. The assembled clamping device used to hold a set of 12 Phytostrips in place so that they can be inverted and sealed at their base with adhesive film. Figure S2. 3-D drawing showing a cross-section of one of the plate-holders. Figure S3. Custom-made fingers for the robotic gripper. Figure S4. Images showing the effect of a range of IAA concentrations on root and shoot development and the reproducibility of the seedling phenotypes across each Phytostrip. [file 13007_2017_158_MOESM1_ESM.docx]

**Additional File 1**

(showing three custom-made devices - see Additional File 2 for the engineering drawings)

**Figure S1**. The assembled clamping device used to hold a set of 12 Phytostrips in place so that they can be inverted and sealed at their base with adhesive film (see Fig. 1). The two identical halves of the clamping device were made in acrylonitrile butadiene styrene (ABS) thermoplastic using a Dimension 1200es 3-D printer (Stratasys, USA).


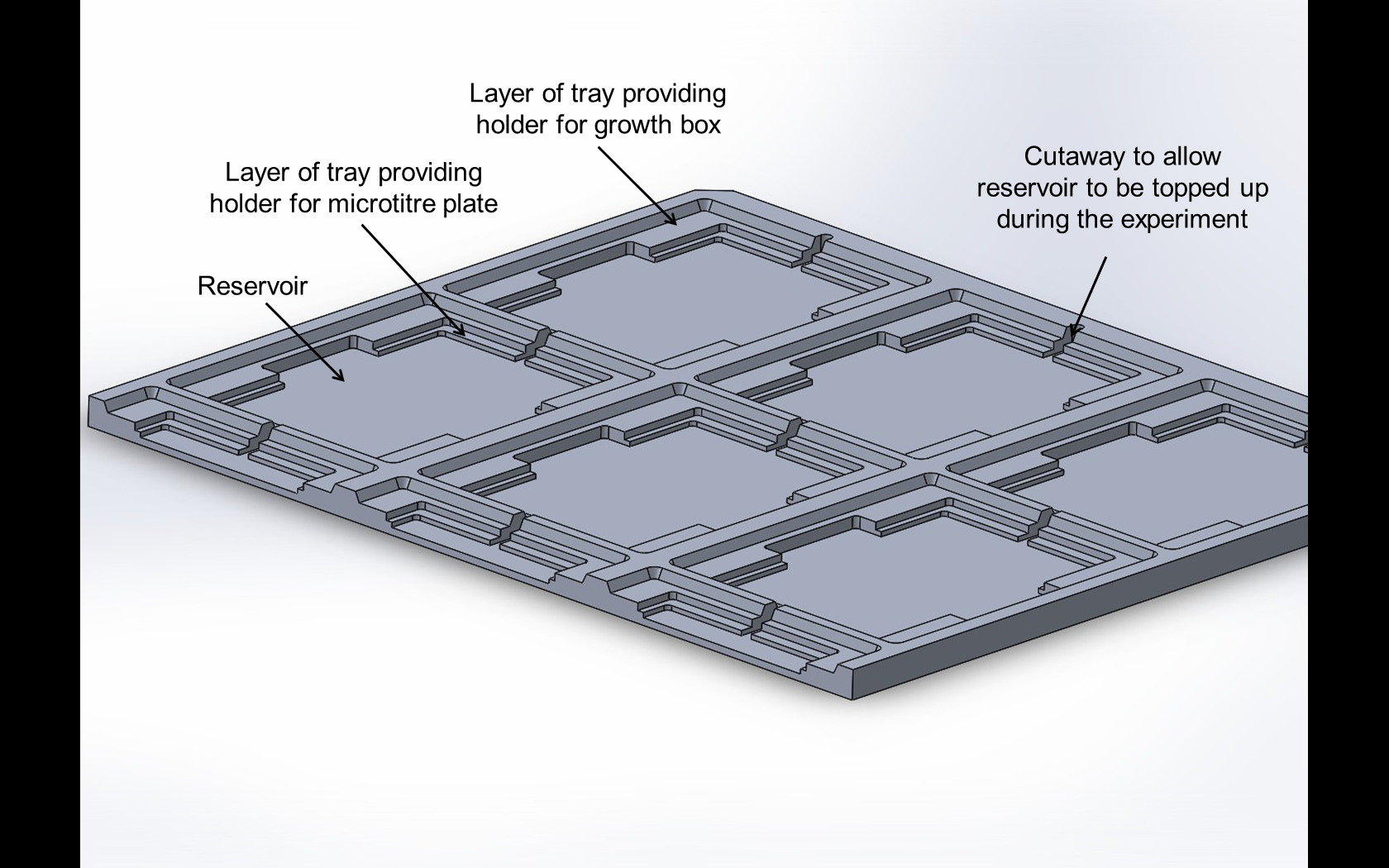


**Figure S2.** 3-D drawing showing a cross-section of one of the plate-holders. Each plate-holder holds 9 assay plates (one assay plate = a microtitre plate + 12 Phytostrips) in a 3 x 3 array and the plate-holders themselves occupy fixed positions on the robotic plinth. The plate-holders are formed in three layers, with the deepest layer providing a reservoir for water to maintain humidity around the assay plates. The second layer provides a microtitre plate-sized holder for the assay plate and the third layer a holder for the plastic growth boxes. The cutaways (arrowed) allow extra water to be added to the reservoir without having to lift the plastic box and also provide for gaseous diffusion between the inside of the growth box and the outside.

**Figure S3.** Custom-made fingers for the robotic gripper. The aluminium fingers are dual-purpose, serving both to remove the plastic growth boxes and to pick up the individual Phytostrips for imaging. **a** 3-D drawing showing two views of the left finger. **b** Close-up of the left finger attached to the robotic gripper. Note the silicone rubber insert which is glued to the inside of the finger to provide a soft grip for lifting the plastic box. An engineering drawing of the left finger is provided in Additional File 2 (the right finger is a mirror image of the left).

**Figure S4.** Images showing the effect of a range of IAA concentrations on root and shoot development and the reproducibility of the seedling phenotypes across each Phytostrip. IAA treatments were applied by diffusion from the microtitre plate wells, beginning on the first day that roots were visible: **a** Control. **b** 12 nM IAA. **c** 37 nM IAA. **d** 110 nM IAA. **e** 330 nM IAA. Note that these are the hypothetical final concentrations that would be present in the Phytostrips once diffusion has gone to completion. Images from above (top panels) and the side (lower panels) were captured 5 d after the IAA treatments were initiated when the seedlings were 7 d old.
