# Supplementary material for: The Microphenotron: a robotic miniaturized plant phenotyping platform with diverse applications in chemical biology
Source: Plant Methods. 2017 Mar 1;13:10. doi: 10.1186/s13007-017-0158-6 (PMC5333401; doi:10.1186/s13007-017-0158-6)
Supplement: Supplementary file 2 — Additional file 2. Engineering drawings of the clamping device, robotic fingers and plate-holder. [file 13007_2017_158_MOESM2_ESM.pdf]

# Clamping device

(two of these needed to make the complete device)

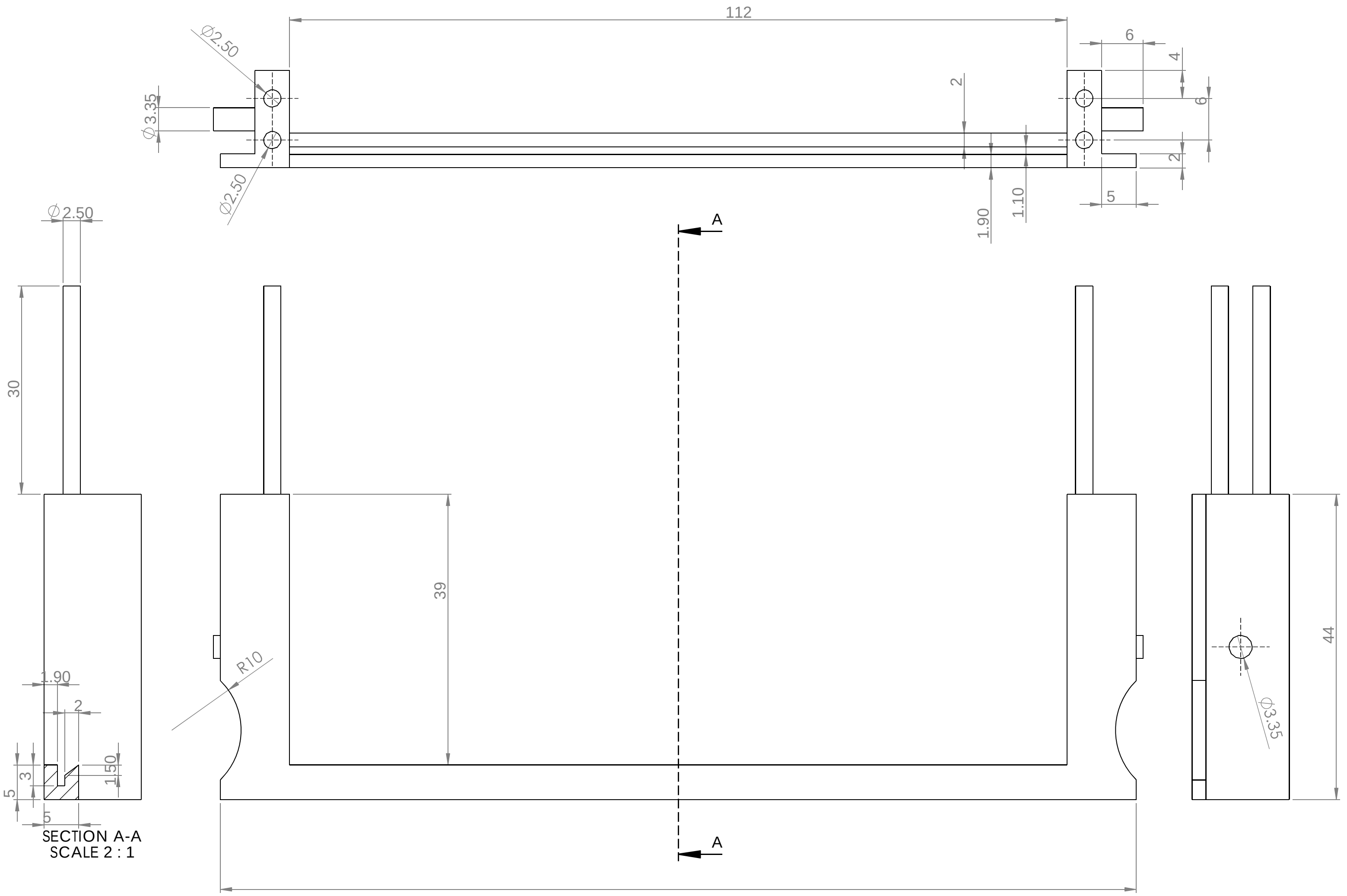

# Left finger

(Right finger is a mirror image of this)

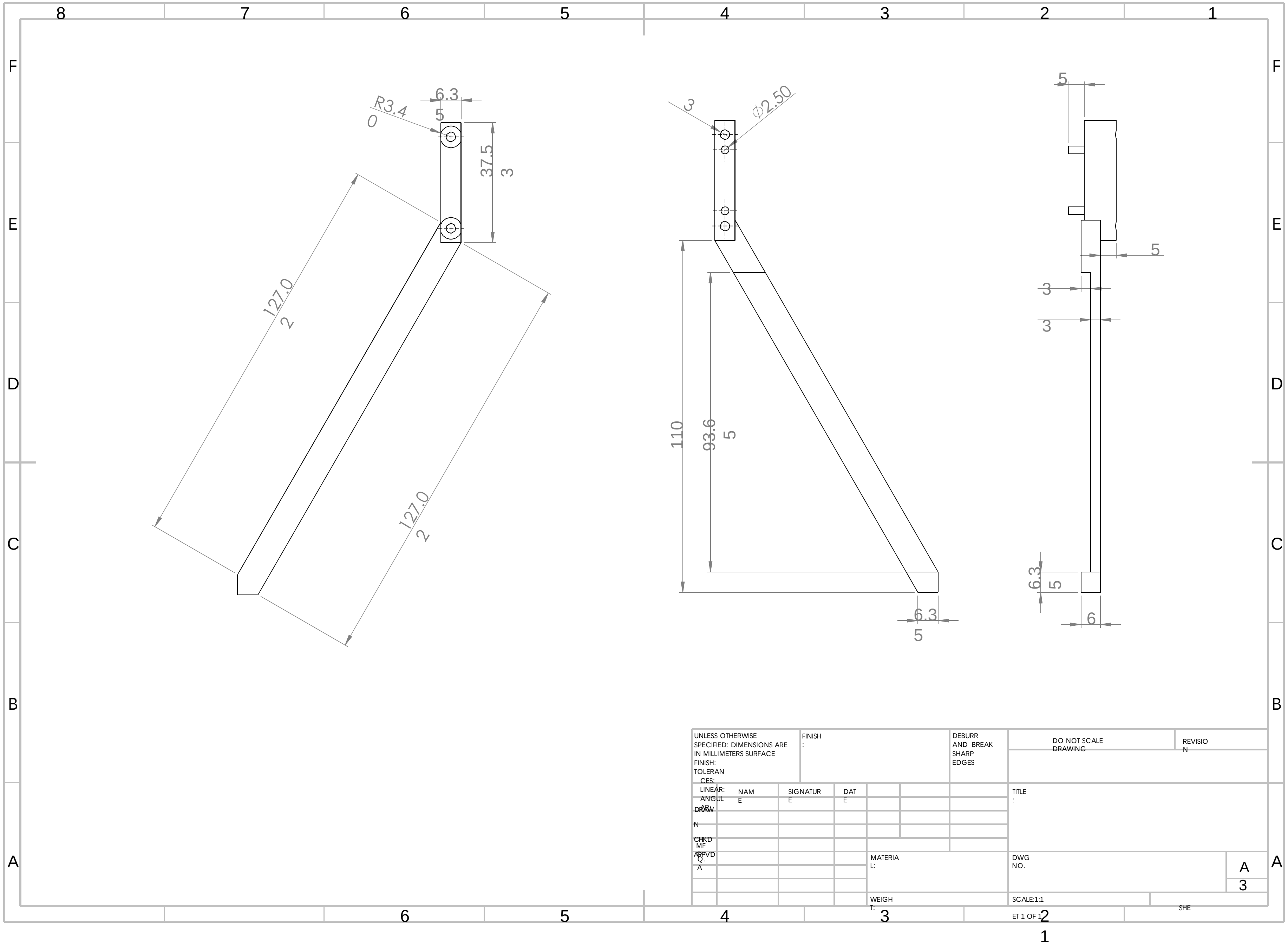

# Plate-holder

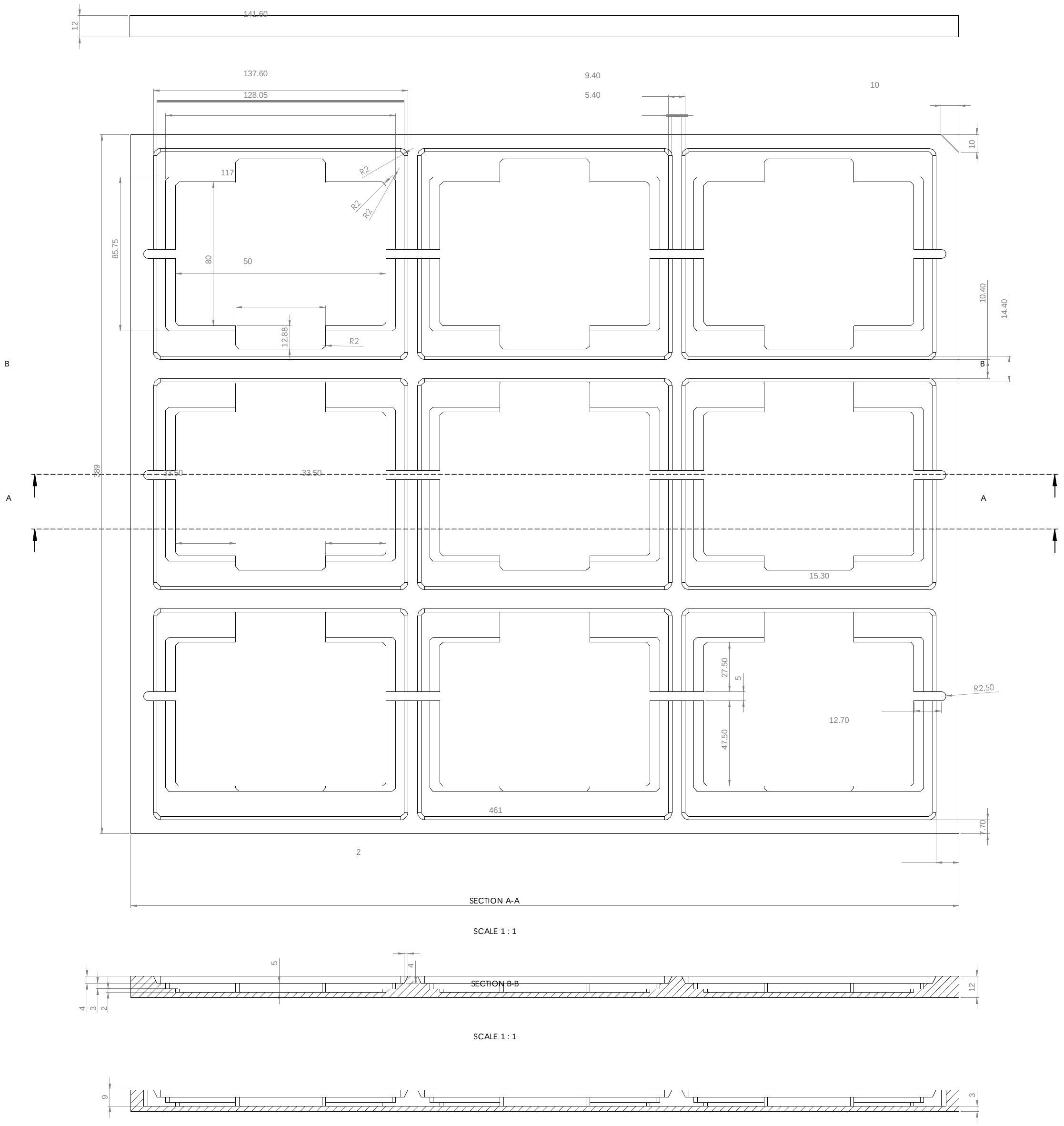


UNLESS OTHERWISE SPECIFIED:
DIMENSIONS ARE IN
MILLIMETERS SURFACE FINISH:
TOLERANCES
: LINEAR:
ANGULAR:

FINISH:

DEBUR AND
BREAK SHARP
EDGES

DO NOT SCALE DRAWING

REVISION

| | NAME | SIGNATURE | DATE |
|---|---|---|---|
| DRAWN | | | |
| CHK'D | | | |
| APPV'D | | | |
| MFG | | | |
| Q.A | | | |

MATERIAL:

TITLE:

late-holder

WEIGHT:

SCALE:1:2

SHEET 1 OF 1
