# Supplementary material for: The Microphenotron: a robotic miniaturized plant phenotyping platform with diverse applications in chemical biology
Source: Plant Methods. 2017 Mar 1;13:10. doi: 10.1186/s13007-017-0158-6 (PMC5333401; doi:10.1186/s13007-017-0158-6)
Supplement: Supplementary file 4 — Additional file 4. A description of the custom control software for the robotic manipulator. [file 13007_2017_158_MOESM4_ESM.docx]

**Additional File 4: A description of the custom control software for the robotic manipulator**

Custom software has been developed that controls both the movements of the robotic manipulator and the settings and actions of the cameras. The same program is also responsible for the user interface.

**Explanation of the programmed sequence of robotic movements.** The robotic manipulator is required to (a) remove all three plastic boxes from one column of assay plates and place them in the drop-off area at the end of the plinth (note that it is necessary to remove all three boxes from the column being sampled to allow the robotic fingers unobstructed access to the Phytostrips) (b) pick up each Phytostrip in turn, present it to the imaging station for image capture and return it to its original location and (c) replace the plastic boxes. The sequence of robotic movements that achieve this is built around two types of movement, absolute and relative. An absolute movement takes the robot to a known position, whilst a relative movement moves the robot by an increment from its current position. The experimental sequence we have devised is designed to be as flexible as possible, so that it can be changed by non-expert users if necessary. For example, to image the Phytostrips in assay plates A1-A3 (see Fig. S4), the sequence begins by moving the robot to an absolute position above plate A3, the gripper lifts the box off this plate and places it at another absolute position in the drop-off zone. It subsequently comes back to the known position above A3. A relative movement takes it forward to plate A2 and the box is removed from here too. The process is repeated for the box at A1. Another relative move takes the gripper to the first Phytostrip in A1. The Phytostrip is lifted and taken to the imaging station (another absolute position) *via* a safe path. The overhead and side images are captured by the cameras and downloaded to the controlling PC. The Phytostrip is returned to its previous position *via* the reverse path. Repeated relative movements take the robot to each strip in the plate before moving on to plate A2. Once the robot has completed imaging of plate A3, it goes back to replace the boxes. This process is repeated for every column as required by the operator. To reduce the build-up of errors, the third position of each plate-holder is defined as an absolute position. Between each plate-holder, the robot moves to the absolute position to regain precision. The sequence algorithm takes the form of a nested ‘for’ loop with internal loops for each plate-holder, each column, and each plate. As a result, it is possible to make straightforward changes to the code without having to alter multiple sections. If the layout or positioning of plate-holders, or even the design of the plate-holders themselves is altered, then the entire sequence can be recalibrated by changing just a number of values on the graphical user interface (GUI). The entire sequence is run within its own thread, and in this manner the rest of the GUI is still active while the sequence is running and other functions can still be activated. This also allows the sequence to be paused without losing any progress.

**Robot Interface.** The robot is connected by Ethernet cable to an Elmo–Gold Maestro motion controller, which is in turn connected to a PC. The Elmo–Gold Maestro is an intelligent multi-axis motion controller with algorithms to control the position of each axis as well as blending the motion for multiple axes moving at once. The manufacturers of the robot provide a .NET Dynamic Link Library (DLL) along with wrappers and instructions to create a custom Visual Basic .NET program which was used to develop software to integrate camera control and to provide a bespoke user interface. Control of the gripper is also integrated into the .DLL provided by PAA. Using the DLL an object of the class ProNEDx is created. Any methods are then applied to this object. A summary of the methods used and their implementation is shown in Table S1.

**Table S1. ProNEDx robot commands**

| **Initialise robot** – Performs set up and connection to the robot for use. Inputs are used to control homing. |
| --- |
| Err As Short = ProNEDx.Initialize(HomeMotors As Boolean, SkipIfHomed As Boolean) |
| **Get error message** – Converts a numerical input error code into a readable string explaining the error. |
| ErrorMessage As String = ProNedx.GetErrorCode(Err As Short) |
| **Move robot to absolute** position – Moves each axis of the robot to an absolute position (CommandPosition in millimetres or degrees) which is an array containing the position of each axis. The Velocity and Acceleration are percentages (1-100) of the maximum. And the final option can pause execution of the code until the move is completed. |
| Err As Short = ProNEDx.MoveAbsoluteAllAxes( CommandPosition As Double, Velocity As Double, Acceleration As Double, WaitForDone As Boolean) |
| **Open Gripper** – Opens the gripper at a percentage of the maximum velocity. The opening position is set in a .txt parameter file. |
| Err As Short = ProNEDx.ServoGripperOpen(Velocity As Double) |
| **Close Gripper** – Closes the gripper at a percentage of the maximum velocity. The closed position is set in a .txt parameter file. |
| Err As Short = ProNEDx.ServoGripperClose(Velocity As Double) |
| **Get the position of single axis** – Saves the position of the selected Axis into a Position variable. |
| Err As Short = ProNEDx.MotorGetCurrentPosition(Axis As Short, Position As Double) |
| **Shut down robot** – Safely closes the connection to the robot. |
| ProNEDx.ShutDown() |

**Camera Interface.** Canon has published the EOS digital software development kit (EDSDK), which is a library of functions for interfacing with EOS digital cameras (https://www.didp.canon-europa.com/). The sample code was modified to support multiple cameras. When the software loads it finds the two Canon cameras connected to the PC, these are selected and they are attached to a controller class. Subsequently, each individual controller can be used to send commands to individual cameras. Relevant commands are listed in Table S2.

**Table S2. EOS digital camera commands**

| **Set Property** – When the Action string is ‘set’, then the selected Property will be set to the value of Option |
| --- |
| Controller.actionPerformed(Action As String, Property As Short, Option As Short) |
| **Take Picture** – When the action string is set to ‘takepicture’ then the camera will be triggered to take a picture that will be downloaded to a predefined location. |
| Controller.actionPerformed(Action As String) |

**User interface.** A graphical user interface has been developed (Fig. S5) to give a non-expert flexible control over the experimental sequence without having to alter source code The GUI contains three tabs that allow the user to control the sequence, calibrate the positions, and calibrate the cameras. Along with user inputs, other useful information is displayed, such as the current position of the robot (both physical and the distance through the experiment), and a system log.

*Settings:* The GUI allows the user to change settings for both the robot and the camera. Firstly the user is able to set a master velocity for the robot. This is a percentage of the maximum speed of the robot. It is also possible to change the speed of the robot in the movement command; in this case, the two percentages are multiplied to produce a slower speed. It is also possible to alter the open and closed positions of the gripper. All three of these commands write new values into a .txt parameter file for the robot, and the user must save the values before they will take effect. The user can also alter the position calibrations, which is a list of all absolute and relative positions discussed above. The positions are defined in the robot’s co-ordinate frame, so the simplest way to obtain these is to manually move the robot there and then record the position. This can be achieved with the manufacturer’s Teach Pendant software which allows the axes to be ‘jogged’. Relative movements are usually the distance between two parts of the system, so these can be measured and aligned with the appropriate axis. Again these must be saved by the user by pressing a button. Finally the user has control over the camera calibration settings (Av, Tv, ISO, metering mode, output quality, and download directory). Relevant settings are saved by the user into a parameter .txt file. Upon start up, all settings are retrieved from this file and pushed back to the cameras in case these have been manually altered.

*Sequence controls.* The user begins by *initialising* the robot, which creates the connection to the robot and passes all parameters to the motion controller. Initialisation is not performed automatically on start-up to keep the robot safe until it is needed. The user then has just three main controls over the robotic sequence: *start*, *pause*, and *stop*. Selecting ‘start’ makes the robot move to the first plate and then perform the full sequence. Selecting ‘pause’ stops the robot after it completes its current move; the sequence starts from its present position if the ‘start’ button is pressed again. Selecting ‘stop’ will stop the robot after its next move and reset the progress. The ‘stop’ button is not a safety stop as the robot will keep moving until it completes its current command. The four controls described are all that are needed to run the experiment in the majority of cases. A final control over the sequence is the ability to select which assay plates are included in the experimental sequence. This is done by clicking on any of the plates in the visualisation of the plate layout (Fig. S5). Assay plates selected to be imaged are shown as green in the GUI and plates not to be imaged are shown as red.

*System log.* This log displays custom system messages that record errors, track user inputs, and provide a record of potentially useful information, especially for development or diagnostic purposes. Each message is time-stamped and at the end of any session, the text in the log is appended to the end of a .txt file for review of any problems.
